# Teaching public health ethics

**DOI:** 10.1186/s40985-015-0007-y

**Published:** 2015-11-05

**Authors:** Christopher Potter

**Affiliations:** Cardiff University, Cardiff, UK

**Keywords:** Teaching, Public health, Ethics, Values

## Abstract

Teaching public health ethics has been recognised as patchy and somewhat theoretically incoherent for some years. Despite Beauchamp and Childress’ work being widely known and used within health care its various principles have been criticised by a number of writers for, among other things, their inherent cultural relativism. This article suggests how a broader ethically oriented approach to the ever-changing field of public health might be pursued – so that a professional public health practitioner might be prepared for a lifelong career of ethical activism. Informed by the pedagogic philosophy of Freire it assumes the development of a module within an MPH programme. If, as is widely acknowledged, we are dealing with essentially a corpus of values, the challenge for an educator of public health ethics is how do we inculcate those values effectively and efficiently? How do we prepare students for as yet unrecognised ethical problems that they might confront? How do we assess such teaching? We know that even in clinical practice gaps appear between accepted ethical principles and actual practice. Within public health the conflicts generated by rival claimants and the contradictions of applying principles at a societal level become even clearer as action to control a situation might restrict the rights of others. This makes it even more important that students are exposed to learning experiences which will equip them adequately for a lifetime of practice. Traditional didactic approaches need to be supplemented with additional modalities such as case studies and role playing. Such case studies might encompass macro policy issues or micro operational issues. Other approaches could include citizen juries, examining international agreements and codes of practice, debates and encouraging explicit reflective practices. The importance of repetition, teachers as role models, courses which themselves demonstrate ethical practice, and a variety of methods are emphasized.

## Introduction

In 1998 Anthony Kessel sent a questionnaire to UK medical institutions enquiring in depth about public health education. In his paper reporting the results he concludes that “there is a strong need for both more education in public health ethics and also further examination of the ethical dimensions of public health” [1]. The patchy teaching of a subject for which “there is no coherent theoretical foundation underpinning” and which largely depended on the bio-medically oriented “principlism” framework of Beauchamp and Childress [2] and an approach based on topics such as public health law, HIV/AIDS and ethics, genetics and research, and so forth, has perhaps changed little despite more journals and books being available to us.

In this article the Beauchamp and Childress framework remains an important starting point for analysing topics and issues such as screening, health promotion, environment, rationing and professional conduct, but the orientation is more towards what Kessel, following Kass, describes as “exploration of ethics in public health” [3]. As Kessel reports, principlism has come under criticism from various quarters for *inter alia*. Its culturally relative Western ‘rationality’ and its suitability for relatively simple problems encountered in clinical research and practice. The health ethicist, David Seedhouse, for example, states that “Beauchamp and Childress have got it lamentably wrong. Nebulous principles generally acceptable to well-heeled Western liberals do no more than offer conclusions (a) open to wide interpretation and (b) acceptable only to those who agree with them in the first place” [4]. He points out that “if you are a Marxist, or a Sartrian, or a utilitarian, or a different sort of deontologist, or a cultural relativist (to name but a few) you will have an insurmountable difficulty in accepting the four principles because they will not be part of the moral theory you espouse.” (Seedhouse [4] p. 130). Finding the four principles “manifestly empty” Seedhouse then offers his own ethical grid as an analytical tool.

Despite the criticism, Beauchamp and Childress’ work is widely known and used within health care and pragmatically we can use it to start raising questions of ethics with students. Similarly, we could use Seedhouse’s ethical grid to analyse situations and cases. However, rather than merely applying principlism or the ethical grid to public health issues, or suggesting a curriculum based on topic areas, this paper attempts to suggest how a broader ethically oriented approach to the ever changing field of public health might be pursued – and in particular how a professional public health practitioner might be prepared for a lifelong career of ethical activism. As Seedhouse emphasises throughout his book “ethics is a complex field, not merely a means of deciding between clear-cut rights and wrongs,” the process of on-going deliberation is core to it (Seedhouse [4] 2002: 45). It is teaching to achieve this end which is the target here.

The author should also perhaps declare the influence of Freire on his own ‘teaching’ practice [5]. Although first published in 1970 and written in the context of experts and peasants Freire’s argument is so often still true of students and their teachers everywhere – especially students coming from traditions (disciplinary or cultural) where the emphasis is on learning facts by rote. “They call themselves ignorant and say the “professor” is the one who has knowledge and to whom they should listen. The criteria of knowledge imposed on them are the conventional ones… Almost never do they realize that they, too, “know things” they have learned in their relations with the world and with other women and men” (Freire [5] 1993:45). In the prologue of his Canterbury Tales Geoffrey Chaucer described the Clerk as: “gladly would he learn, and gladly teach” (Chaucer [6] p. 11). That seems a good model for those trying to work with public health practitioners, whether actual or potential, on the ethics of a discipline which is forever expanding and coping with a growingly complex world.

Seedhouse describes how when he first started “there was no room for ethics in official curricula, and the meetings I was asked to lead were squeezed into lunchtime slots or stitched into other courses…” (Seedhouse [4] 2002:1). It may well be that readers will be faced with the same challenge. Public health ethics might be a module on an undergraduate or postgraduate course, or it might be a one-off lecture or seminar, or just ‘stitched into’ other modules. Students might be full-time or part-time, or perhaps not following a structured programme of study but merely health professionals wanting to be brought up to speed on the topic. For the purposes of this article it is assumed that we are considering a module of some sort devoted to the topic on, perhaps, an MPH programme, and that the tutor is a public health specialist with an informed knowledge of the subject rather than a professional ethicist. What is offered must be adapted according to the realities of the situation.

## Review

### “The business of philosophy is not just to understand the world but to change it” Marx

#### The nature of public health ethics

Unless you believe in a revelatory deity who dictates behavioural standards and values we must accept that ethics are based on value judgements and that these are culturally determined. They are relative and they will change over time. Even those who wish to refer back to a revelation (Jews, Christians, Muslims, Church of the Latter Day Saints), or the insights and codes of great leaders and sages (Sikhs, Buddhists, Daoists, Confuscianists, deontologists following Kant), will recognise that the cultural and temporal milieus dominant at the time they originated mean that words must be reinterpreted for application in new conditions. However clear a principle or precept may seem (“thou shalt not kill” or “*primum non nocere,*” for example, widely used since it appeared in 1860, ascribed to Thomas Sydenham, 1624 – 1689 [7]) in practice we must balance competing interests and sometimes conflicting values so that we choose a “lesser evil.” The great religions all have their caches of interpretations to sit alongside the original holy books (Talmud, hadiths, canon law) just as common law has grown up alongside legal codes showing how the original statements could or should be understood.

That public health ethics are based on values was openly acknowledged by Kass [3]. “A framework of ethics analysis geared specifically for public health is needed, both to provide practical guidance for public health professionals and to highlight the defining values of public health, values that differ in morally relevant ways from values that define clinical practice and research” (Kass [3] 2001:1776). If we are dealing with essentially a corpus of values the challenge for an educator or trainer of public health ethics is how do we inculcate those values effectively and efficiently? There is a knowledge component, but because ethical values only have value to society when put into practice, we need to know how those being trained or educated will recognise that they are confronting an ethical challenge, that they will be able to interpret their knowledge of values appropriately to cope with what might be a novel situation in society, and that they respond with appropriate behaviour – possibly under circumstances when powerful interests wish them to behave otherwise (managers, a noisy element of the public, a military tyrant). Finally, for the educationalist, we must ask how we can assess how well the student has taken on board the material.

Given that there may be considerable gaps in time between learning about ethics and being confronted with an issue containing ethical considerations we must be preparing the student in a way that has a lasting effect. Although they might overlap education and training are not the same thing. As Albert Einstein is reported to have said “Education is what remains after one has forgotten what one has learned in school” [8]. We increasingly live in a technological age where the information and skills we are taught are obsolete within a few years. It would appear that by its very nature ethics requires more than training, and that we are well into the realm of education. This raises its own set of philosophical questions. Do we try and teach – or try to provide a learning opportunity for students to teach themselves? If values are to be applied how do we provide opportunities for students to practise skills of interpretation and application so that they become ingrained responses to daily problem solving for years to come?

It is unrealistic to expect that the educator takes responsibility for whether a student later chooses to follow an ethical course of action or not. However, it is not unreasonable to ask the educator to ensure that the student has been exposed to relevant material so that he or she appreciates the ethical issues and the values involved, and also that they have demonstrated some sort of ability to use that material in a variety of situations.

Undoubtedly, most health care practitioners have been more systematically exposed to ethical training than were previous generations. The monumental work of Beauchamp and Childress [2] has provided a readily available text book which has become widely used despite the criticism it has attracted as described above. It deals in depth with the various moral approaches underpinning biomedical ethics, and presents its well known framework of four fundamental principles: respect for autonomy, nonmaleficence, beneficence and justice. It also shows how such values can lead to contradictory outcomes. The work grew out of a milieu of concerns about technology, the role of the State in health care delivery and the dialogue about rights that grew up after two world wars. Some of the growing concern about health ethics that has arisen in the last half a century was directly sparked by research and practices indulged in by the Axis powers during World War II; research such as the infamous Tuskegee Syphilis Study described by Tulchinsky and Varavikova [9]; the pace of technological changes and their sometimes unfortunate outcomes (e.g., the use of thalidomide); post-modern scepticism about science, authority and the way problems are presented; recognition of the social determinants of health, and, therefore, perceived nanny-state interference in the way people live; and growing litigation in some countries so that health professionals and their employers had to justify their actions.

As Kass acknowledges, “much of public health is inherently and unabashedly paternalistic” (Kass [3] 2001:1778) and there are legitimate concerns about, say, polypharmacy, collection of information, water fluoridisation, controls over personal behaviour and general “nanny-state-ism.” Those of a collectivist orientation who believe they are on the side of the angels are likely to brush aside the concerns about restricting liberty. “The health of the people is the highest law” they proclaim, quoting (usually without realising it) Cicero, and almost certainly forgetting that the eloquent orator was eventually executed with his hands and tongue pinned on a wall for public view. They also forget that the phrase could be twisted to justify eugenic experiments, and, within the lifetime of many still alive today, to validate ‘euthanasia’ of the mentally handicapped and mass execution of, for example, Jews, homosexuals, those of mixed race, and Romas. As Tulchinsky and Varavikova [9] point out: American eugenics policies were praised by Hitler and these ideas were adopted in Nazi Germany leading to execution of half a million ‘undesirables’ under the eugenics concept, and were adapted for mass extermination of the Jews, Gypsies, and others in the holocaust” (Tulchinsky and Varavikova 2009:597). More detailed analyses of how eugenics concepts crossed the Atlantic and influenced the Third Reich in Germany, how “social hygiene” and the “mercy killing” of babies developed into the even more monstrous atrocities performed in the pursuit of “racial hygiene,” and how some medical staff became willing practitioners of these atrocities can be found in the collection of papers edited by Nicosia and Huener [10] and Ulf Schmidt’s biography of Karl Brandt [11].

So health professionals coming into public health will probably already be aware of the need to behave ethically. Furthermore, in Western countries, at least, research will come under the scrutiny of ethics committees so many students will have encountered ethical challenges.

#### Teaching, training or learning?

It is relatively easy to *teach* people the various approaches to ethics which have been formulated in the past (the virtue ethics of the classical period; principle driven approaches such as utilitarianism; deontological or duty based as advocated by Kant; or Beauchamp and Childress’ framework). We can provide the names and dates of the great exponents and thinkers. We can show how the ideas could be applied, and describe their strengths and weaknesses. We can also explain how the law and ethics might interact, how “rights” and principles might create dilemmas and consider various examples of how ethical issues were created by technological or political changes. This material is readily available in text books and on-line. Students can learn it like they learn the names of bones and muscles and can be tested on how well they have assimilated facts in the period available.

Many of the professionals who come into public health will already have been given some sort of training in ethics and will have a code of practice (doctors, pharmacists, nurses, some of whom will have taken the Hippocratic Oath, see Tulchinsky and Varavikova [9] 2005:116); others may need to start from basics. However, we only need to look around the world to see how poorly many individual health care practitioners live up to the ethical codes they profess. Patients are exploited, the most needy often neglected; resources are used for research or patterns of care that fail to match up with the health needs of the majority; counterfeit drugs are peddled and medicines prescribed inappropriately; managerial performance standards are allowed to interfere with safe and evidence based care provision; female genital mutilation and torture are still being carried out. Sometimes practitioners have simply not recognised the ethical issues involved or have failed to see that societal values have changed so that they withhold information required for proper consent or perpetuate discrimination.

If this has been somewhat problematic for clinical ethics it poses a much greater challenge for the growing field of public health ethics. As other articles will have described, we are not merely considering how we relate to a patient or small group of patients but on how laws might be passed that affect the whole population or even groups of countries. We are restricting the rights of some to protect the many. We are coping with how resources should be transferred to benefit populations while knowing there will be winners and losers both at the population and individual level. We are advising on the use or non-use of community-wide vaccination, screening, food additives and drug use (statins, fluoride, anti-virals) knowing that society statistically may benefit but actual flesh and blood individuals may suffer side effects, including premature death, they would not otherwise have experienced. We are consulted about new technologies to avoid and old ones to abandon, knowing that individuals might benefit although society as a whole cannot justify investment. We are managers and planners advising on resource investment for uncertain demographic and environmental futures. The conflicts generated by rival claimants and the contradictions of applying principles at a societal level become clearer than at the individual level. Even the practices used to implement public health policy, and even to analyse public health policy, may raise ethical issues. As Buse, Mays and Walt [12] observe, policy analysis raises ethical issues about who is invited to participate in coalitions, how far information may be withheld for tactical advantage, how far are we comfortable compromising to “accommodate and win over a policy opponent?” (Buse, Mays and Walt [12] 2005:190). And it is frequently carried out in a very public arena, where pressure is applied by pharmaceutical companies, politicians, activist groups, the press, senior managers and possibly even military forces.

The inadequacies of current approaches to biomedical ethics have grown more apparent over the years, as accompanying articles have demonstrated, and we have benefitted from specific papers and books on public health ethics [3, 13, 14]; as well as books on public health oriented issues which explicitly raise ethical concerns [15–17].

#### Providing a learning experience

Ethics as a branch of philosophy has been taught since at least the time of Socrates and Plato, and formed part of the classical education curriculum over the centuries. So we might assume that there is plenty to guide us in how to teach the subject. In fact until relatively recently teaching consisted of didactic lectures, self-learning through reading, and perhaps small group tutorials talking through issues arising from the reading students had undertaken and essays they had written. In recent years universities have made greater use of case studies to challenge students, whereby individual students or groups consider the case presented and write a paper or make a presentation on the issues arising. Although there may be a “right answer” better cases will have been designed to create conflicts and the need for compromises, thereby reflecting more accurately real-life situations students may face in their place of practice. Variations on this will require students to role play advocates for particular positions or to hold debates between teams, to try and bring out the inherently value based nature of ethics.

In so far as public health ethics is being taught as an abstract subject it might be sufficient to rely on traditional didactic teaching methods. References, handouts, talk-and-chalk presentations, essays, and exams mean we can expose students to the ideas, names and dates to test their capacity to remember, apply and communicate them. In fact, time permitting, there is a case for some such material because it is interesting in its own right, and traditional methods remain an efficient way of transferring a large body of information. If, on the other hand, we wish to ensure our students will take away skills for life that enable them to apply ethical considerations to their real life public health practice, including situations we may not even be able to imagine at present, then we must use additional approaches. Indeed, if we wish our students to incorporate an ethical dimension into their own practice - and even to become activists changing society for the ‘better’ - we must go much further.

One place to start would be with case studies and role playing to assist students to engage with ideas and principles which will need application in real life situations. The emphasis moves from “teaching” to “learning.” Cases may be generated by the teaching staff, but students can be involved further by encouraging them to identify possible ethical concerns from their own professional experience, current work (if part-timers), the experience of relatives and friends, current issues or emerging technologies as reported in the press. Cases are increasingly available on line and CDC Atlanta have been compiling a set of cases which educators can access (the student handbook is a free download and teachers can request the facilitators’ handbook) [18].

If teachers (or ‘learning facilitators’) prefer to develop their own cases there is no shortage of issues which could be used to construct realistic scenarios. There are macro issues about corporate capture of policy making concerning pharmaceuticals, food, tobacco and alcohol or in partnership working. Whether public health professionals (whose role is to protect the public) should accept posts as civil servants (whose primary *raison d’être* is to protect the minister), and if they are employed in such posts how do they balance competing demands? How far should statistics be massaged or presented selectively to serve political ends or public health ends we believe in but where the evidence is less than convincing? At the other end of the spectrum there are micro issues such as operational activities to contain an outbreak where we must advise on prophylactic antibiotics or vaccination (with attendant risks) for someone who is not ill themselves, or to close a petting farm (with its economic consequences for the owners and staff) while further investigations are taking place. Kant’s challenge that we should not use people as means to an end is a constant challenge for us as public health professionals.

By raising such matters in group settings it will usually quickly become evident that there are a range of values held by participants, and they will vary in the weight they put on the Beauchamp and Childress principles, for example. Some will be more concerned with autonomy and liberty, others with collective good outcomes, some with avoiding maleficence, others more with promoting beneficence. It will become evident that participants interpret civic virtue differently. For example, some participants might balance the rights of the unborn against those of the mother or potential siblings differently. The debate that follows will usually generate not just facts but affect which helps with memorisation of issues and sensitises participants to alternative perspectives in ways which will linger far longer than facts they have read or memorised.

We are not starting from first principles each time we design a public health intervention, and a variety of codes of practice have been articulated to guide the way different aspects of public health practitioners should design health promotion or health development projects. Examples include:

- Helsinki Declaration (1964) by the World Medical Association on clinical research [19].
- Ottawa Charter for Health Promotion (1986*)..which “put health on the agenda of policy-makers in all sectors and at all levels…”* (Tulchinsky and Varavikova [9] 2009:41–42)
- Cairo Population Conference. Program of Action led by UNFPA (1994) [20].
- Ljubljana Charter on Reforming Health Care (1996) – Europe only [21].
- Johannesburg Declaration on Sustainable Development (2002) [22].
- Oslo Ministerial Declaration on Global Health (2007) [23].
- Paris Declaration on Aid Effectiveness, 2005 (56 commitments on ownership, accountability, harmonisation, etc.) and Accra Agenda for Action (2008) [24].
- Venice Statement (2009) on global health initiatives and health systems [25].
- Rio Political Declaration on Social Determinants of Health (2011) [26].

Although these and similar declarations can be taught by traditional means, they are more usefully taught by encouraging students to think through how well they address ethical challenges, how far they are observed, to what extent they reflect particular cultural, temporal or political points of view. Students can be encouraged to consider hypothetical public health projects and interventions to assess how congruent they are with these declarations.

Another way students might be encouraged to reflect on ethical dimensions of public health problems is to design a way of introducing a public health intervention which would engender broad support or validation despite it requiring the weighting of a range of values which might be in tension. For example how might they constitute a citizens’ jury to consider the matter? Who would be invited to serve? What questions, witnesses and evidence might need exploring? Would a set of focus groups be more appropriate to gauge the range of values and brought into play by the proposed intervention?

Moving on from that position, how might students use the reaction of a citizens’ jury or focus group to promulgate or introduce an intervention which would run counter to, say, the majority preference or the views of vested interests but would promote an ethically sounder public health outcome for a minority group or future generations? What would be the ethical issues surrounding such a strategy? The aim all the time being to make the student realise that however partisan they may be for or against a position, and however strong they think the evidence may be, there are values, and therefore, ethical dimensions which must be considered.

Through the use of such exercises one is striving to ensure such a reflective response becomes second nature to the public health practitioner, so that they remain sensitive to the rights and needs of all concerned even if ultimately hard decisions have to be made. There is a need to prevent the tunnel vision which passion for a position can create; or where one might be lulled into believing that experiments or worse should be carried out in the name of eugenics, cost-effectiveness or political mandates. Triage decisions in the aftermath of a disaster or during a catastrophic epidemic might introduce all sorts of ethical considerations such as occurred during the SARS pandemic in 2003 and the Ebola epidemic in West Africa in 2014–15 for example. Far better to have thought through such positions in the study or classroom beforehand, rather than fail to recognise them or make short-term decisions under pressure which are later regretted. Increasingly decisions, whether made under pressure or as part of new policies regarding health system design or health policy, will be scrutinized, and even if the right ethical decisions were made one may need to provide a justification. Again, better to make the decision with a clear recognition of the ethical dimensions and how and why the final position was reached.

As Lindert and Potter [27] suggest in the accompanying article on methodology of learning modules development, in helping students reflect on situations and cases from a public health ethics perspective it is useful to take as a starting point the question *“Qui bono?”* or “Who benefits?” from the current situation or would benefit from the proposed changes or intervention. Who wields what power and in what ways? This might be brought out through role playing scenarios.

#### Instilling a lifelong ethical practice

As suggested above, we can seek to address public health ethics at several different levels. If it is merely ensuring students have been exposed to the topic as an academic exercise then a few lectures and maybe a couple of case studies is sufficient. If we want public health practitioners for whom an ethical awareness and sensitivity is second nature then the learning experience must inculcate ethical reflection as a habit. Within training and education there are at least two ways this can be accomplished. One is intensity of experience, something the student is unlikely to forget, the other is repetition. The intensity aspect was referred to earlier through the debates and group work, particularly with heterogeneous groups (discipline, nationality, sex, age, and so on) which hopefully will expose students to a wider range of values they have been aware of previously. Additionally, students can be asked to adopt a position through role playing or genuinely working through a case study or situation, then facing challenges from colleagues brought in from outside the department, for example professional ethicists, representatives from patient groups, local politicians or senior policy makers who can ask challenging questions.

Repetition can simply be a series of case studies and seminars over a longer period. Alternately, or in addition, students can be encouraged to keep a diary or log book where they record issues which arise in their day to day practice (for part-time students) or as they read journals and newspapers, watch TV or talk to friends and colleagues. Has a new health technology been announced? Has a health authority faced litigation? Is a reconfiguration of health services being proposed locally? Have religious leaders or politicians publicly declared concerns about some aspect of health or social care? Are politicians or public health colleagues elsewhere reported as contemplating new legislation to ban an activity, promote new health interventions or introduce rationing (however it might be dressed up)? Are pressure groups representing a disease group demanding their “rights”? Are commercial interests resisting or promoting changes to food/fuel/tobacco/alcohol/medicines standards, packaging, or prices? Students should note such events and ask themselves what ethical issues are involved. Who is benefitting from the *status quo*? Who will benefit from the changes? Who will lose? Is it really the public who will gain, or is this being done to satisfy the needs of health professionals themselves, or their managers, or politicians? Should public health professionals take a position? How might they take action? Such log books increase sensitivity in public health practitioners until it becomes second nature. The aim is not to lead to paralysis by analysis, and practitioners may wish to push through a measure despite some people’s rights or cherished values being violated – but it will be done reflectively and purposefully and may even improve their strategies and tactics for accomplishing beneficial public health change despite powerful opposition. Staff teaching ethics can also do this and such logbooks are an excellent source for teaching examples and case study development.

Another way repetition can be employed is by ensuring colleagues teaching other modules on public health programmes, such a global health, health economics, health policy, epidemiological research methods, regularly identify, or better still encourage students to identify, ethical aspects of the material they are teaching. An ethical discussion can also be a requirement in dissertation preparation. An ethical awareness should be part of the warp and woof of public health education, and such an approach helps prevent students thinking of ethics as contained in a separate academic box. It is encouraging to see recent specialised textbooks for undergraduates explicitly addressing ethics [9, 12, 28].

It perhaps goes without saying that students through the ages have been enthused by their educators, taking teachers as role models. The educator who takes the lead role on a public health programme for teaching ethics must be an enthusiast and must themselves demonstrate that they have an ongoing interest in applying ethical analysis to public health practice. But it is also the wider faculty who have a responsibility to display a commitment to ethical public health practice and show that it is not an optional extra.

It is also essential that the programme itself is run on ethical lines, with systems to ensure that staff and students are recruited without discrimination; assignments are fairly distributed and marked; harassment and bullying are eliminated. Public Health teaching accreditation as promoted by ASPHER and APHEA examine such issues and by accreditation institutions demonstrate their commitment to quality and ethical practice.

#### Need to adapt methods to student needs

Not everyone learns the same way. Some people prefer didactic teaching, some prefer a more hands-on approach. Some are self-disciplined and will readily read and reflect on material, others need a structure and motivation. Various models of learning have been generated over the years. A well-known one is Kolb’s model which he first published in 1984 and has been re-developed and elaborated by Kolb and many others since. It can be readily accessed on the web. See for example www.businessballs for diagrams of the cycle which he described [29]. Questionnaires are available which show whether we are, to use one example, activists, reflectors, theorists or pragmatists [30]. Students who are activists and pragmatists might find public health ethics itself somewhat akin to debating how many angels can sit on a pin and the educator must try and develop methods and exercises which will spark their interest and keep them motivated. Students who are reflectors and theorists may become so bogged down in the ideas and analysis that they neglect the “so what?” dimension. In the types of learning approaches described above there is sufficient mix that students of all learning preference should be able to find something that will not just expose them to ideas and facts, but will encourage lifelong reflective and activist public health practice.

Where public health ethics is being taught as part of a formal programme some sort of assessment of students will almost certainly be required. Besides the traditional examination paper using essays multi-choice question and short answers educators might consider more adventurous approaches, for example, requiring students to respond to a case study and marking their analysis and conclusions or developing a role playing exercise. In both these approaches they could be working as individuals or groups and be faced with a critical outsider who will challenge their position. Their log book identifying ethical issues over several months could be assessed, and where students are part-timers they could be required to prepare reflective papers on aspects of their current work showing how they have incorporated ethical analysis in their practice.

## Conclusion

Finally, although this article has been written mainly with an academic, fixed duration public health programme in mind, such as an MPH, we do want life-long reflection and practice. Some groups of public health practitioners, or even individuals, may want to deliberately expose themselves to ethical challenge by engaging with a mentor with an ethical commitment (perhaps a professional philosopher/ethicist) to reflect on the ethical dimensions of their work. The keeping of a diary or logbook as described above would give material for sessions with such a mentor or group events run like a journal club to encourage ethical reflection and practice. Being part of such an arrangement could help an isolated practitioner who feels pressured while making resource investment decisions, for example, maintain an ethical perspective (nowadays sessions can be run via the internet not only face to face). It could also be used to count towards formal continuous professional development, where this required.
